# The Defect of iNKT Cells Mediates Mitochondrial Dysfunction Through IL-6/STAT3 Signaling to Induce Atrial Inflammation and Fibrosis to Aggravate Postoperative Atrial Fibrillation

**DOI:** 10.33549/physiolres.935803

**Published:** 2026-08-01

**Authors:** Xiao TIAN, Guangshu TIAN, Zhishuo LIU, Lihua LV, Lin XIA, Zhonglu YANG

**Affiliations:** 1 Department of Anesthesiology, General Hospital of Northern Theater Command, Shenyang City, Liaoning Province, China; 2 Department of Cardiovascular Surgery, General Hospital of Northern Theater Command, Shenyang City, Liaoning Province, China

**Keywords:** iNKT cells, Atrial fibrillation, Inflammation, Mitochondria, IL-6/STAT3 pathway

## Abstract

Atrial inflammation and fibrosis are the pathogenesis of postoperative atrial fibrillation (POAF). It has been reported that invariant natural killer T (iNKT) cells can coordinate tissue inflammation, but whether it can regulate POAF is still unknown.

A sterile pericarditis (SP) model was constructed on C57BL/6J mice and Jα18^−/−^ mice to simulate POAF. Intraperitoneal injection of anti-interleukin (IL) -6 neutralizing antibody (anti-IL-6) was used for intervention, and atrial fibrillation (AF) was induced by transesophageal sinus atrial node pulse pacing. The levels of iNKT cells and IL-6/signal transducer and activator of transcription 3 (STAT3) signaling pathway were detected by flow cytometry and western blot. HE stain, Masson stain, and immunohistochemistry were used to evaluate the process of inflammation and fibrosis in atrial tissue. The calcium homeostasis and function of atrial mitochondria were evaluated by biochemical detection and fluorescent staining. The expression of iNKT cells was up-regulated in SP mice. The defect of iNKT cells increased the AF induction rate and duration of SP mice, enhanced AF susceptibility, aggravated atrial electrical remodeling, promoted inflammatory cell infiltration and fibrosis, and increased inflammatory factors and fibrotic proteins. Compared with SP mice, iNKT cell defects also aggravated endoplasmic reticulum-mitochondrial calcium coupling disorder, mitochondrial calcium homeostasis imbalance, reduced mitochondrial DNA (mtDNA), adenosine triphosphate (ATP) content, and mitochondrial complex enzyme activity, and increased IL-6 and STAT3 phosphorylation levels. Anti-IL-6 significantly inhibited IL-6/STAT3 signaling pathway, reduced AF induction rate and duration, and improved atrial mitochondrial calcium homeostasis, mitochondrial dysfunction, inflammation, and fibrosis. The deficiency of iNKT cells aggravated atrial tissue mitochondrial dysfunction, inflammation, and fibrosis in POAF mice via activating IL-6/STAT3 axis, and aggravated AF susceptibility.

## Graphical abstract

The deficiency of iNKT cells can aggravate the imbalance of mitochondrial calcium homeostasis and mitochondrial damage in POAF *via* activating IL-6/STAT3 axis, aggravate inflammatory cell infiltration and fibrosis in atrial tissue, and increase the susceptibility to AF.

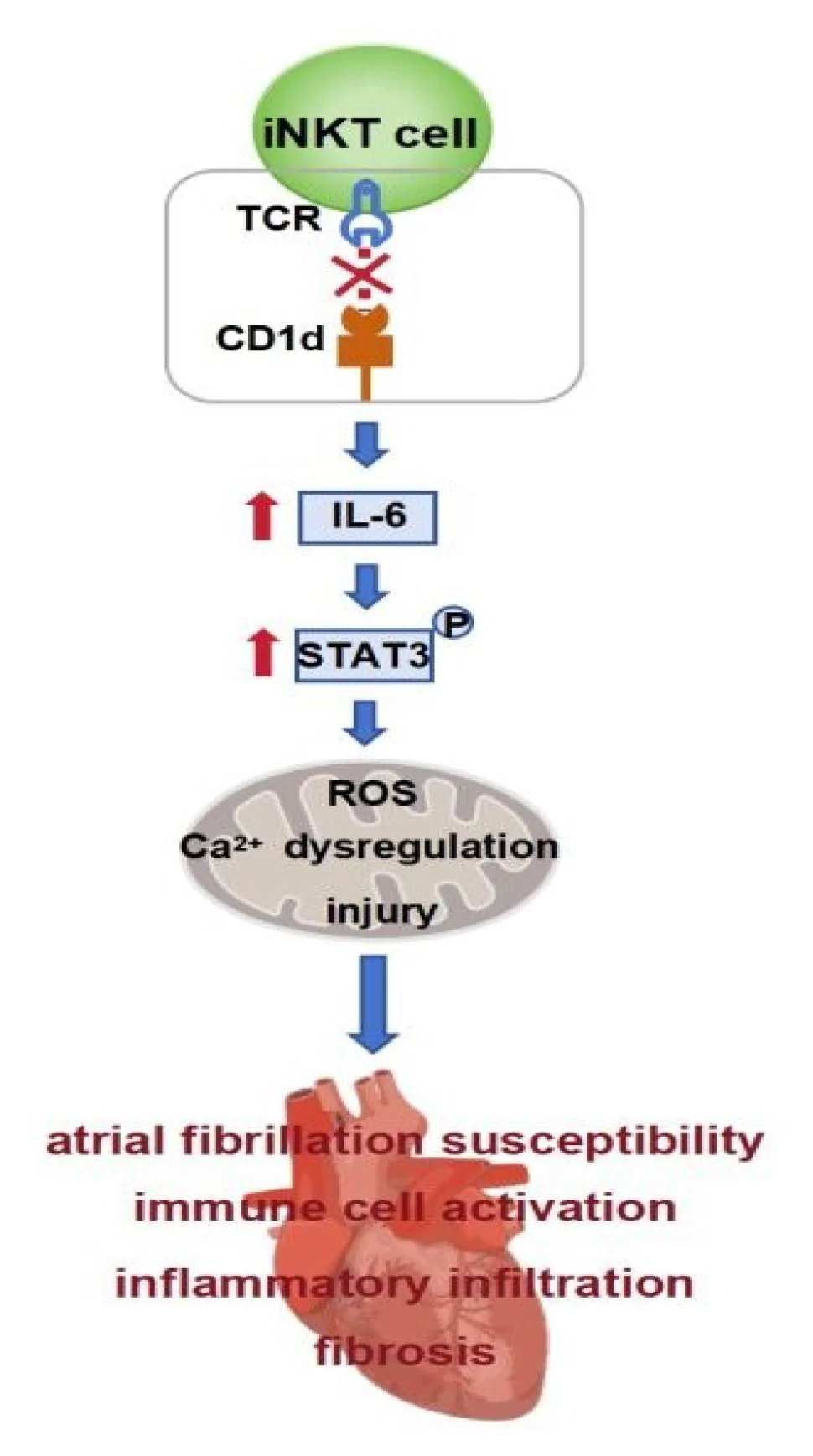

## Introduction

Postoperative atrial fibrillation (POAF) is a new onset of atrial fibrillation (AF) within a period of time after surgery (common in cardiac valve surgery, cardiac bypass grafting, etc.), affecting 30 %–50 % of patients [1]. It is generally believed that POAF is a common secondary AF, which not only leads to hemodynamic disorders, enhances complication risks like heart failure and stroke, but also prolongs hospitalization time, increases medical costs, and even affects the long-term survival rate after surgery [2, 3]. However, POAF precise pathogenesis remains incompletely understood, and there is a lack of effective prevention strategies. Therefore, it is very important to explore the pathophysiological process of POAF.

A large amount of evidence shows that the occurrence of AF results from the interaction of many factors, the main influencing factors are cardiac electrical remodeling and structural remodeling. Among them, structural remodeling plays a pivotal role in AF progression, especially the pathological changes characterized by atrial inflammation and fibrosis, which are the key matrix for AF occurrence and maintenance [1, 4]. Therefore, reducing inflammation and myocardial fibrosis is an effective means to prevent and treat AF [5, 6]. In addition, mitochondria are essential organelles in eukaryotes and are a source of energy for cells [7]. Many pathophysiological processes of AF are related to mitochondrial function, such as energy metabolism disorders, oxidative stress, and calcium homeostasis. In physiological cardiomyocytes, mitochondria produce adenosine triphosphate (ATP) through tricarboxylic acid cycle (TAC) and oxidative phosphorylation, which maintains the function and enzyme activity of ion channels, ion pumps, and transporters in the cell membrane and endoplasmic reticulum, and provides energy for the mechanical and electrical activities of the heart [8]. Disruption of mitochondrial calcium homeostasis contributes to intracellular Ca^2+^ overload, which in turn further impairs mitochondrial function, ultimately leading to reactive oxygen species (ROS) accumulation and oxidative stress injury. This subsequently triggers mitochondrial dysfunction, Ca^2+^ leakage, and cardiomyocyte apoptosis, and promotes AF maintenance [9]. Studies have also found that mitochondrial dysfunction is an emerging key mechanism driving cardiac inflammation and fibrosis, and alleviating atrial inflammation and fibrosis by reducing mitochondrial dysfunction is a possible AF treatment method [10].

Natural killer T (NKT) cells are a special heterogeneous subset of lymphocytes that express both T cell receptor (TCR) and natural killer cell (NK) cell receptor on their surface [11]. According to whether NKT cells express constant TCR, NKT cells are divided into two categories: type I NKT cells express constant TCR, also known as invariant NKT (iNKT) [12]; type II NKT cells express a diverse TCR repertoire and are therefore difficult to identify [13]. The iNKT cells were activated after specific recognition of glycolipid antigens presented by cluster of differentiation 1d (CD1d). Activated iNKT cells can rapidly produce various cytokines (interferon-γ (IFN-γ), interleukin (IL)-4, tumor necrosis factor (TNF), IL-17, and IL-10, etc.) to coordinate tissue inflammation [14]. Compared with non-arrhythmia patients and healthy people, the proportion of NKT cells in AF patients was higher [15]. And there are NK cells in the pericardial space after surgery [16]. Single-cell RNA sequencing analysis showed that CD8 T cells and NK cells in AF clots showed up-regulated genes related to stress response, extracellular matrix remodeling, and immune regulation [17]. Therefore, iNKT cells may affect AF by regulating the inflammatory response.

However, the specific role of iNKT cells in the development of POAF and its potential mechanism are still unclear. Based on the above research background, we propose the following scientific hypothesis: iNKT cell deficiency increases atrial inflammation, fibrosis, and mitochondrial dysfunction by activating the IL-6/STAT3 signaling pathway, thereby increasing POAF susceptibility. Therefore, in this study, a mouse model of sterile pericarditis (SP) was established to simulate the pathogenesis of POAF, to study atrial tissue inflammation, fibrosis, mitochondrial damage and AF susceptibility, and to systematically evaluate iNKT cell involvement in POAF occurrence and its molecular mechanism, intending to offer reference and experimental bases for iNKT cell therapy development and iNKT cell clinical trials.

## Methods

### POAF mouse model

Male C57BL/6J wild-type (WT) mice, 6–8 weeks old, weighing 18–22 g, purchased from Ziyuan Experimental Animal Technology Co., Ltd. (Hangzhou, China); 6–8-week-old male Traj18 gene knockout (Jα18^−/−^) mice (KO, iNKT cell defect model), weighing 18–22 g, purchased from Southern Model Biotechnology Co., Ltd. (Shanghai, China). The Traj18 gene encoded the invariant α-chain (Vα14-Jα18) of the TCR specifically expressed by iNKT cells. This chain was essential for iNKT cells to recognize lipid antigens presented by CD1d. Consequently, Jα18^−/−^ mice failed to generate a functional TCR for iNKT cells, leading to a developmental block at the thymic stage and a near-complete absence of iNKT cells in peripheral lymphoid organs and tissues. This model was therefore widely recognized as a classic tool for investigating the functional roles of iNKT cells [18, 19]. To avoid the potential interference of estrogen on the experimental outcomes, only male mice were used in this study [20]. All mice were housed in an SPF animal room with a light/dark cycle of 12 h, a temperature of 23 °C, and a relative humidity of 50 %. This experiment was approved by the General Hospital of Northern Theater Command Ethics Committee.

SP mouse model was established [21]: mice were anesthetized by intraperitoneal injection of 1 % pentobarbital sodium solution (40 mg/kg). After the reflex activity of the limbs was weakened, it was fixed on the small animal operating table for tracheal intubation and ventilator-assisted breathing. After skin preparation and disinfection in the precordium of the mouse, skin incision, blunt separation of chest muscles, and opening of the chest cavity were performed in order to expose the heart. The pericardium of the atrial area was torn open with ophthalmic forceps, and sterile talcum powder was evenly applied to both sides of the atrium. The chest cavity was closed, the skin was sutured, and disinfected again. After the recovery of respiratory function, the ventilator was removed. The experimental groups were as follows: Sham group (n=7): only thoracotomy was performed, the pericardium was not torn open, and talcum powder was not applied; SP group (n=7): SP surgery was performed.

SP mice were intraperitoneally injected with 50 μg of neutralizing antibody goat anti-mouse IL-6 mAb (BE0046, Bio X Cell, USA) or normal goat IgG (BE0087, Bio X Cell). Antibodies were administered at 36 h after SP induction, and heart tissues of each group were collected 12 h later.

**Fig. 1:**
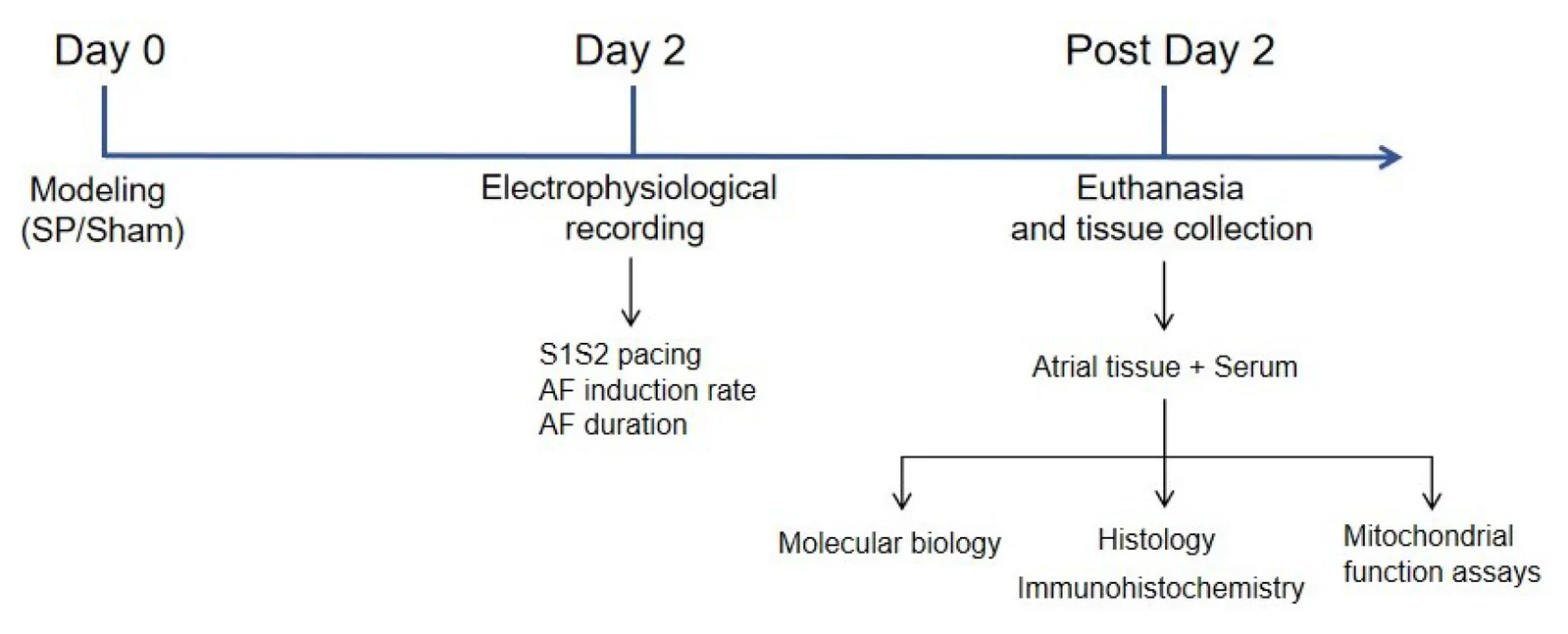
Flow chart of experiment

After electrical stimulation of the mouse heart, the eyeball was removed for blood collection, and centrifuged to separate serum for subsequent experiments. Mouse atrial tissue was quickly removed, part of which was stored in liquid nitrogen or PBS, and part of which was fixed in 4 % paraformaldehyde. The detailed experimental process was shown in Fig. 1.

### Programmed electrical stimulation

The mice were placed in a small animal anesthesia machine (R500, Rivard, Shenzhen, China). After isoflurane inhalation anesthesia, the mice were fixed on the small animal fixation plate (supine position), and endotracheal intubation was performed through the mouth. The small animal ventilator (tidal volume 1.2 mL/min, respiratory ratio 5 : 4) was connected. The electrodes were connected to the mouse limbs and connected to the electrocardiogram (420 s physiological recorder, Taimeng, Chengdu, China). The RR (the time between two consecutive R wave peaks, ms), P wave duration (ms), PR (the time from the starting point of the P wave to the starting point of the QRS wave, ms) and QRS (the time from the starting point of the QRS wave to the end point, ms) were recorded stably. All parameters were averaged from three consecutive heart beats to improve accuracy.

The mice underwent thoracotomy to expose the heart, tear the pericardium, and install the stimulation electrode. The stimulator was set to 'overdrive pacing' mode (basic cycle length = 100 ms), and the ventricle was rapidly paced. SNRT100 (the time interval between the last pacing stimulation signal and the first spontaneous sinus P wave, ms) and AERP100 (the longest S1–S2 pairing interval causing atrial depolarization, ms) were recorded. The measurement were repeated three times in the same part to ensure the stability of the results.

AF was induced by programmed electrical stimulation (S1S2 stimulation) on postoperative day 2. The stimulation protocol consisted of 30 S1 stimuli (basic cycle length = 100 ms) followed by a single premature S2 stimulus. The S2 coupling interval was progressively decreased from 45 ms to 35 ms in 2 ms decrements. Successful AF induction was defined as a rapid, irregular atrial rhythm lasting at least 1 second. If AF occurred at least once in two pacing trials, the induction of AF was considered positive. The overall AF burden was calculated by summing the durations of all individual AF episodes. The AF induction rate was derived by dividing the number of AF episodes by the total number of procedures performed.

### Flow cytometry experiment

Fresh atrial tissue preserved in PBS was washed with PBS and cut into small pieces of tissue fragments, which were incubated with 5 mL Liberase TM (LIBTM-RO, Sigma Aldrich, USA) in dark for 30 min. After digestion, an equal volume of precooled DMEM containing 10 % fatal bovine serum (FBS) was added to terminate reactions. Filter the mixture with 200 mesh nylon gauze. The liquid was collected into a flow tube and centrifuged to collect cell pellet. An appropriate amount of PBS was added to re-suspend the cells to prepare a single cell suspension. Single cell suspension was stained with CD45 Percp, CD3 PI, NK1.1 APC (NKT cell marker), CD45 FITC, CD1d PI (CD1d positive leukocyte marker), CD69 (iNKT cell activation marker) antibodies, and their homologous isotype-matched negative controls. Then flow cytometry analysis was carried out, and lymphocyte subsets were preliminarily identified by forward scattering (FSC) and lateral scattering (SSC). The lymphocyte population was accurately screened by the parameter scattering map (CD45/SSC) in the flow cytometry, and other cell debris were excluded. Finally, CD1d^+^CD45^+^, CD45^+^CD3^+^NK1.1^+^, and CD69^+^ cell subsets were specifically identified in the selected CD45^+^ lymphocyte population.

### Western blot

0.05 g atrial tissue was weighed and 0.6 mL of freshly prepared and pre-cooled lysate was added. The tissue was ground and crushed by a freeze grinding machine, and the supernatant was obtained after lysis and centrifugation. The supernatant was total protein. These concentrations in atrial tissue were measured by the BCA method. After the protein denaturation operation was completed, the protein was separated by 10 % SDS-PAGE gel electrophoresis, and then the protein was transferred to the PVDF membrane at a current of 200 mA. The 5 % skim milk powder solution was prepared with TBST, and the PVDF membrane was placed in it. Under normal temperature conditions, the PVDF membrane was slowly shaken and incubated on a shaker for 2 h, and then the PVDF membrane was washed three times on a rapid shaker using TBST, 10 min each time. Finally, they were incubated with the primary antibody at 4 °C for about 14 h. The corresponding secondary antibody was added and incubated for 1 h. The ECL chemiluminescence kit was used for development, and the gel imaging system (ChemiDoc MP, Bio-Rad, USA) was used for imaging and photographing. ImageJ software analyzed the gray value of band. The primary antibodies are shown in Table 1.

### HE staining and Masson staining

The mouse atrial tissue was fixed for 24 h, followed by gradient ethanol dehydration, xylene transparent treatment, and paraffin embedding. Tissue section 4 μm thick was made by a paraffin sectioning machine (HistoCoreArcadia H+C, Leica, Germany). The sections were first dewaxed with xylene, then hydrated with gradient ethanol, then stained with hematoxylin for 5 min, washed with running water to return to blue, stained with eosin for 2 min, and finally sealed with neutral gum. The cell morphology, inflammatory cell infiltration, and tissue arrangement were observed by microscope (BX53, Olympus, Japan), and the pathological changes of atrial fibrosis were analyzed.

After dewaxing and hydration, the paraffin sections were immersed in Weigert hematoxylin staining solution for 10 min, ponceau acid fuchsin solution for 5 min, phosphomolybdic acid solution for 5 min, and finally stained with aniline blue staining solution for 5 min. The distribution of collagen fibers (blue) and atrial cells (red) was observed, and the area of collagen fibers was quantitatively analyzed by ImageJ software.

### Immunohistochemistry

Paraffin sections of atrial tissue were taken and boiled in antigen repair solution for 20 min. After cooling, endogenous peroxidase was added dropwise and reacted for 10 min. The cells were incubated with myeloperoxidase (MPO, ab188211, 1 : 8 000, Abcam), CD68 (ab303565, 1 : 1 000, Abcam), α-SMA (BF9212, 1 : 200, Affinity), IL-6 (ab290735, 1 : 100, Abcam), and STAT3 (ab109085, 1 : 100, Abcam) primary antibodies overnight at 4 °C. On the next day, add the reaction enhancement solution and incubate for 20 min, and PBST was washed and incubated with secondary antibody for 40 min. Finally, reaction was stained with DAB, terminated with PBST, and stained with hematoxylin for 2 min. Finally, the protein expression was observed under a microscope and analyzed by ImageJ software.

### ELISA

ELISA kits detected the contents of inflammation, fibrosis, and mitochondrial related indicators in each group of mice. The transforming growth factor β1 (TGF-β1) ELISA kit was purchased from BIOESN (BES0038K, Shanghai, China). IL-1β, IL-6, monocyte chemoattractant protein-1 (MCP-1), IL-10, and platelet-derived growth factor AB (PDGF-AB) ELISA kits were purchased from MEIMIAN (MM-0040M1, MM-0163M1, MM-0082M2, MM-0176M1, MM-0140M2, Jiangsu, China). Kits for malondialdehyde (MDA), superoxide dismutase (SOD), ATP, and mitochondrial complex enzyme I, II, and III were purchased from GIVEI (JW.MO1306, JW-G21637, JW-G21590, JW.MO2064, JW.MO2065, JW.MO2066, Shanghai, China). The mouse atrial tissue was added with normal saline and smashed, and the supernatant was taken after centrifugation. The experimental steps were performed in accordance with ELISA kits. The OD value of each hole was detected at 450 nm using a multifunctional microplate reader (Gen5, Bio-Tek, USA), and zeroed with a blank hole. The standard curve was obtained by taking the standard concentration as the abscissa and the OD value as the ordinate, and the sample concentration was calculated by taking the OD value of each group.

### Transmission electron microscopy (TEM) detection

A small piece of atrial tissue (about 1 mm^3^ in volume) was placed in 2 % glutaraldehyde and fixed at 4 °C overnight. After dehydration with different concentrations of ethanol, the ultra-thin sections were cut and stained with 4 % uranyl acetate and 0.4 % lead citrate. Finally, the ultrastructural changes of atrial cells were observed under transmission electron microscope (ARM300F, JEOL Ltd, Japan). For quantitative analysis of mitochondrial morphology, at least five randomly selected fields per sample were captured at ×15,000 magnification, and the following parameters were assessed using Fiji software: mitochondrial number was manually counted per field of view; mitochondrial cristae surface density (Sv, μm^2^/μm^3^) was calculated as the total cristae membrane surface area per unit mitochondrial volume using the point-counting method; and mitochondrial volume density (Vv, %) was calculated as the percentage of mitochondrial area relative to total cytoplasmic area. All quantifications were performed in a blinded manner by an investigator unaware of the experimental groups.

### Rhod-2 AM fluorescent probe

Mitochondrial calcium content was detected by Rhod-2 AM. The atrial tissue was loaded with 5 μmol/L Rhod-2AM and 0.02 % Pluronic F-127 and incubated in dark at 37 °C for 30 min. The tissue was washed with fresh, calcium-containing normal Tyrode's solution for 3–4 times to remove excess probes. The tissue was transferred to fresh Tyrode's solution and incubated for 20–30 min, allowing intracellular esterases to fully hydrolyze Rhod-2 AM into Rhod-2 and enrich it in mitochondria. The loaded atrial tissue was fixed in the imaging bath, and the oxygenated, 37 °C Tyrode's solution was continuously perfused to maintain tissue activity and physiological state. The bath was placed on the fluorescence microscope stage, and the Rhod-2 AM was imaged under the excitation wavelength of 530 nm and the emission wavelength of 576 nm in the scanning mode of Line Scan. A 40× oil-immersion objective lens (numerical aperture 1.3, Olympus, Japan) was used for image acquisition. The 2 Hz field stimulation was applied to the electrode in the bath to record the complete Ca^2+^ transient. ImageJ software was used to analyze the amplitude and rise rate.

### Quantification of mitochondrial DNA (mtDNA) and mitochondrial protein (mtProtein)

According to previous research reports [22], total DNA was extracted according to the instructions of the QIAamp DNA MINI kit (51306, QIAGEN, Germany). The mitochondrial respiratory chain complex I subunit gene NADH dehydrogenase 1 (ND1) was used to express mtDNA, and 18S ribosomal RNA (18S rRNA) was used to express nuclear DNA (nDNA). TB Green Premix EX Taq^TM^ II (Tli RNaseH Plus) kit (RR820A, TAKARA, Japan) was used for qPCR reaction. The levels of mtDNA and nDNA were measured, and the mtDNA content was determined by mtDNA/nDNA ratio. The primers were synthesized by Shanghai Shenggong Bioengineering Co., Ltd. The sequence was as follows: ND1 (F: 5′-ATGGC CAACCTCCTACTCCT-3′, R: 5′-GCGGTGATGTAGA GGGTGAT-3′); 18S rRNA (F: 5′-CATTCGAACGTC TGCCCTATC-3′, R: 5′-CCTGCTGCCTTCCTTGGA-3′).

Atrial mitochondria were isolated using a mitochondrial isolation kit (C3606, Beyotime, Shanghai, China). The atrial tissue was cut into small fragments, and the mitochondrial separation reagent and homogenate were added. The homogenate was centrifuged, the supernatant was centrifuged again, and the pellet was mitochondria. The mitochondrial lysate was added, centrifuged, and the supernatant was collected to extract the mitochondrial protein. The mtProtein concentration was determined by BCA method and normalized to the total protein content.

### DHE staining

The atrial tissue was frozen and sectioned at a thickness of 5 μm. After treatment with 0.3 % Triton X-100 for 10 min, the sections were incubated with 50 μmol/L DHE in dark for 1 h. The nuclei were stained with DAPI. The stained sections were imaged using a fluorescence microscope (CKX53, Olympus, Japan) and the average fluorescence intensity was measured using ImageJ software.

### Statistical analysis

Statistical analysis was conducted using SPSS version 27.0. Normality and homogeneity of variance were assessed for all data sets. One-way ANOVA was utilized, followed by Tukey's post hoc test for group comparisons. Data for each group were presented as mean ± standard deviation, with *P*<0.05 deemed statistically significant.

## Results

### iNKT cells were up-regulated in SP mice

**Fig. 2:**
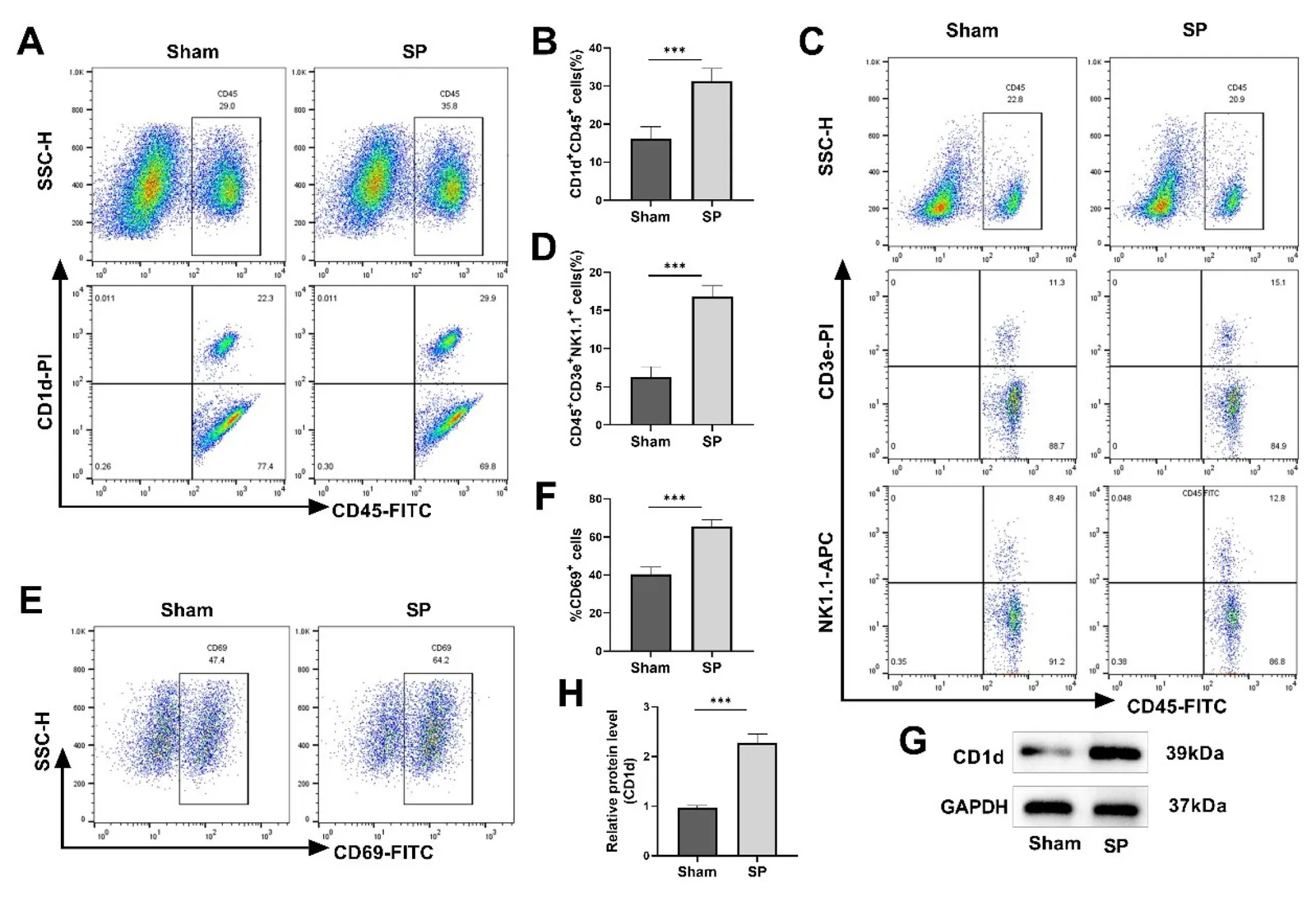
iNKT cells were up-regulated in SP mice. **A–B**) SP mouse model was established, and the expression of CD1d positive leukocytes (CD1d^+^CD45^+^) in atrial tissue was analyzed by flow cytometry, which was significantly increased in SP mice. **C–D**) The expression of iNKT cells (CD45^+^CD3^+^NK1.1^+^) in atrial tissue was detected using flow cytometry, which was markedly enhanced in SP mice. **E–F**) The expression of iNKT cell activation marker CD69 in atrial tissue was analyzed by flow cytometry, which was notably enhanced in SP mice. **G–H**) CD1d protein in atrial tissue was analyzed by western blot, which was significantly increased in SP mice. n=7, ***P < 0.001.

The number of CD1d positive leukocytes (CD1d^+^CD45^+^) and iNKT cells (CD45^+^CD3^+^NK1.1^+^) in atrium of SP mice increased significantly (Fig. 2A–D), and the iNKT cell activation marker CD69^+^ cells also increased significantly (Fig. 2E–F). Western blot data proved that CD1d protein in SP atrial tissues was also markedly increased (Fig. 2G–H).

### iNKT deficiency increased AF susceptibility in SP mice

**Fig. 3:**
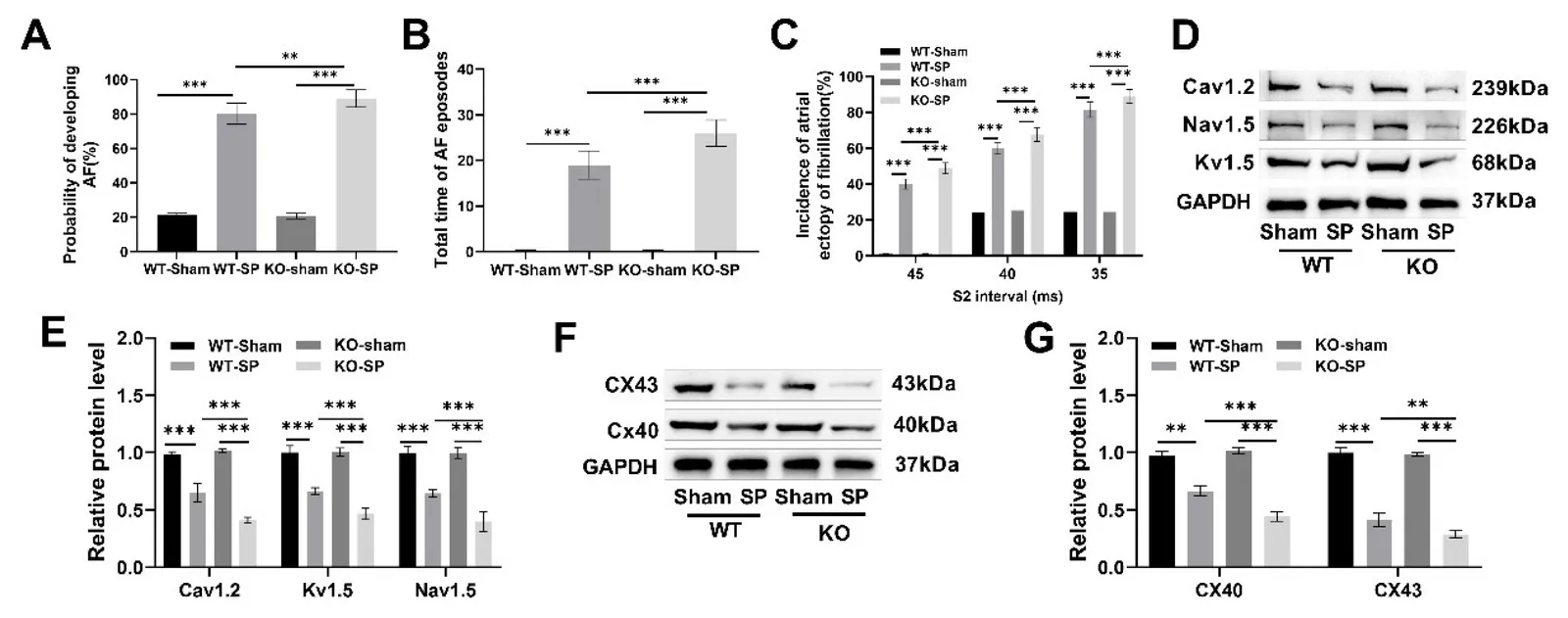
iNKT deficiency increased AF susceptibility in SP mice. **A–B**) The statistical data of the probabilities and durations of AF induced 2 days after surgery were significantly increased in SP mice. **C**) The incidence of atrial ectopic or fibrillation per S2 interval was significantly increased in SP mice. **D–G**) AF-related proteins in atrial tissue were detected using western blot. The levels of Cav1.2, Kv1.5, Nav1.5, CX40, and CX43 proteins were significantly decreased in SP mice. n=7, **P < 0.01, ***P < 0.001.

In this study, the AF susceptibility of SP mice was detected. There was no difference in basic ECG parameters between Sham mice and SP mice, and the data are shown in Table S1. The indexes such as atrial conduction heterogeneity and AF induction rate increased significantly after SP, especially on the 2^nd^–3^rd^ day after operation, which was in line with the clinical pathogenesis of POAF. Therefore, S1S2 stimulation was used to induce AF on the 2nd day after operation. As expected, the incidence of AF in WT-Sham mice was 21.21 %, that in WT-SP mice was 80.29 %, and the total duration was 18.99 seconds. The incidence of AF in KO-Sham mice was 20.69 %, that in KO-SP mice was 89.00 %, and the total duration was 25.98 seconds. The success rates and durations of AF in SP mice were significantly increased, and the success rates and durations of AF in KO-SP mice were markedly higher than those in WT-SP mice (Fig. 3A–B), indicating that iNKT-deficient mice were more prone to induce AF. Similarly, the hearts of WT-SP and KO-SP mice showed more frequent atrial ectopic or fibrillation under the stimulation of S1S2, and the atrial ectopic or fibrillation in KO-SP mice was more obvious (Fig. 3C). This indicated that S1S2 stimulation successfully induced AF in SP mice, while the defect of iNKT cells significantly aggravated AF in SP mice. Cav1.2, Kv1.5, and Nav1.5 were the three most crucial ion channels in cardiac action potentials, and their functional abnormalities are closely related to AF maintenance. CX40 and CX43 were the most important connexins in the heart and form the structural basis for direct electrical and chemical communication between myocardial cells. Cav1.2, Kv1.5, Nav1.5, CX40, and CX43 proteins were markedly decreased in SP mice, and these protein expressions were further decreased in KO-SP mice (Fig. 3D–G), suggesting abnormal electrical conduction in atrial tissue of SP mice. The defect of iNKT cells exacerbated the electrical conduction disorder. The above experiments consistently and strongly prove that the defect of iNKT cells greatly aggravates the electrophysiological remodeling of SP, leading to extremely unstable atrial electrical activity, thereby significantly increasing the susceptibility and persistence of AF.

### iNKT deficiency aggravated inflammatory cell infiltration and fibrosis in atrial tissue of SP mice

**Fig. 4:**
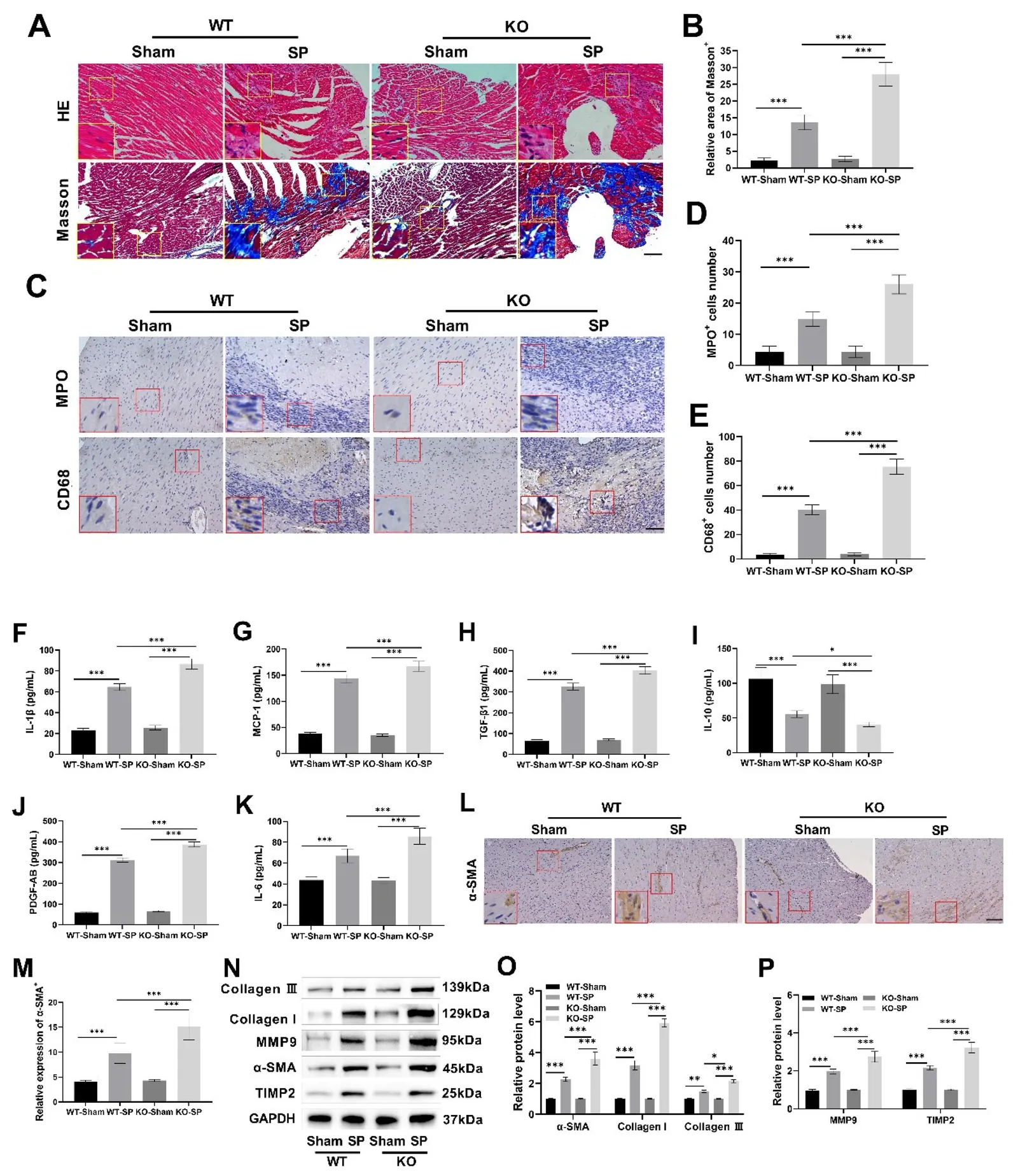
iNKT deficiency aggravated inflammatory cell infiltration and fibrosis in atrial tissue of SP mice**. A–B**) HE and Masson staining detected atrial pathological changes. SP tissues exhibited marked inflammatory cell infiltration and cardiomyocyte necrosis. SP tissues exhibited marked blue collagen deposition (reflecting early fibrotic changes) (×20, 100 μm). **C–E**) Inflammatory cells in atrial tissue were detected by immunohistochemistry. MPO^+^ cell numbers and CD68^+^ cell numbers were significantly enhanced in SP mice (×40, 50 μm). **F–K**) ELISA analyzed inflammatory factors. In SP mice, the contents of IL-1β, IL-6, MCP-1, TGF-β1, and PDGF-AB were also markedly raised, and IL-10 level was notably declined. **L–M**) α-SMA level in atrial tissue was analyzed by immunohistochemistry, which was significantly increased in SP mice (×20, 100 μm). **N–P**) Western blot analyzed fibrosis proteins in atrial tissue. α-SMA, Collagen I, Collagen III, MMP9, and TIMP2 levels were significantly raised in SP mice. n=7, *P < 0.05, **P < 0.01, ***P < 0.001.

Atrial remodeling was considered to be a key factor in AF occurrence and maintenance, and inflammation and myocardial fibrosis are involved in the process of myocardial remodeling [5]. The myocardial tissue structure of Sham mice was complete, the myocardial cells were uniform in size and arranged neatly, showing no significant inflammatory cell infiltration and pathological damage. SP mice myocardial structure was significantly damaged, and the arrangement was disordered. A large number of inflammatory cell infiltration and cytoplasmic dissolution and necrosis of myocardial cells were observed. The myocardial structure of Sham mice was clear, and almost no collagen deposition was observed. In SP mice, obvious blue collagen deposition was observed, and collagen deposition area was significantly increased, Notably, the area of blue collagen deposition in KO-SP mice was even larger (Fig. 4A–B). Immunohistochemical data proved that MPO^+^ cell numbers and CD68^+^ cell numbers in SP atrial tissue increased significantly (Fig.4C–E), suggesting that the number of neutrophils and macrophages increased. The content of IL-1β, IL-6, MCP-1, TGF-β1 and PDGF-AB also enhanced markedly, and IL-10 level decreased significantly (Fig.4F–K). This indicated that inflammatory cell infiltration occurred in the atrial tissue of SP mice, and the inflammatory level of KO-SP mice was markedly higher, and the levels of inflammatory cells and factors were further increased. In addition, immunohistochemical data proved that the positive rate of α-SMA was markedly enhanced in SP atrial tissue (Fig.4L–M), and the protein expressions of α-SMA, Collagen I, Collagen III, MMP9, and TIMP2 were also significantly increased (Fig.4N–P). The atrial tissue of SP mice had obvious fibrosis, and the fibrosis degree of KO-SP mice was markedly higher, and the level of fibrosis protein was further increased. In summary, iNKT cell defects aggravate the degree of inflammation and fibrosis in SP mice.

### iNKT deficiency aggravated mitochondrial calcium homeostasis imbalance and mitochondrial damage in SP mice

**Fig. 5:**
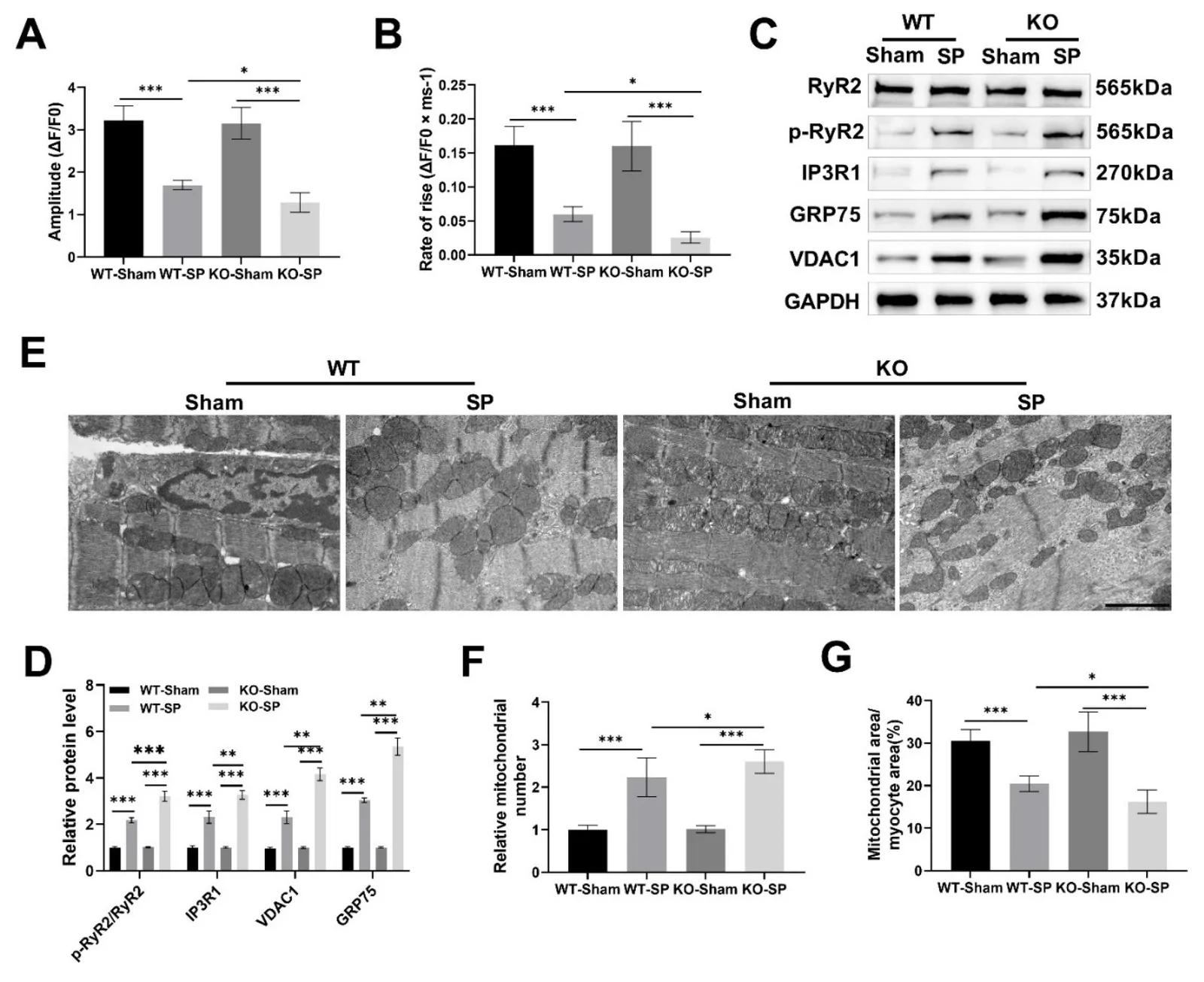

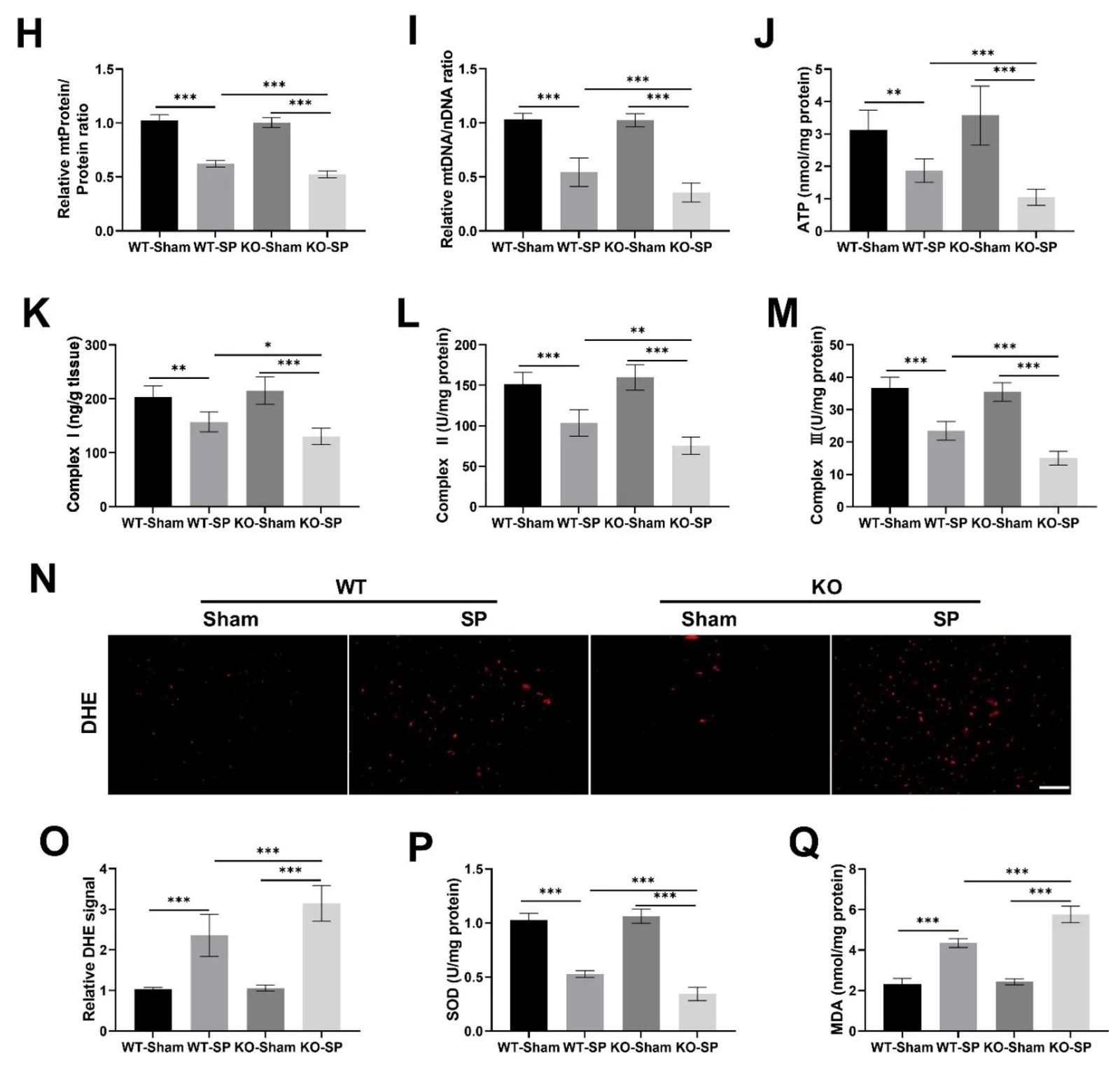
iNKT deficiency aggravated mitochondrial calcium homeostasis imbalance and mitochondrial damage in SP mice**. A–B**) The transient parameters of Ca^2+^ in atrial tissue were calculated. The amplitude and rising rate of Ca^2+^ were significantly decreased in SP mice. **C–D**) Western blot detected mitochondrial-endoplasmic reticulum calcium coupling related proteins. p-RyR2, IP3R1, VDAC1, and GRP75 proteins were markedly increased in SP mice. **E–G**) The morphology of mitochondria in atrial tissue was observed by TEM. The mitochondria of SP mice were swollen, the cristae were reduced or broken, the number of mitochondria was significantly increased, and the surface density of mitochondrial cristae and mitochondrial volume density were significantly decreased (×15.0k, 2.0 μm). **H–I**) The content of mtProtein and mtDNA in atrial tissue was detected, which was significantly decreased in SP mice. **J–M**) ATP content and mitochondrial complex enzyme I, II, III activity in atrial tissue were measured, which were significantly decreased in SP mice. **N–O**) DHE staining assessed atrial ROS levels, which were significantly increased in SP mice (×40, 50 μm). **P–Q**) The content of oxidation index in atrial tissue was detected by kit. The content of MDA in SP mice was markedly enhanced, and SOD content was significantly lessened. n=7, *P < 0.05, **P < 0.01, ***P < 0.001.

This study investigated the effects of iNKT cell deficiency on mitochondrial function in the atrial tissue of SP model mice, and evaluated it from multiple dimensions such as mitochondrial calcium homeostasis, morphology, biosynthesis, function, and oxidative stress. The transient amplitude of mitochondrial Ca^2+^ in SP mice was significantly reduced, the rate of increase was significantly reduced (Fig. 5A–B), and the expression levels of mitochondrial-endoplasmic reticulum calcium coupling-related proteins p-RyR2, IP3R1, VDAC1, and GRP75 were significantly increased. Compared with WT-SP mice, the transient amplitude and rising rate of Ca^2+^ were significantly decreased in KO-SP mice, and the levels of mitochondrial-endoplasmic reticulum calcium coupling-related proteins were significantly increased (Fig.5C–D). These findings suggested endoplasmic reticulum-mitochondrial calcium coupling was enhanced in the atrial tissue of SP mice, which was further aggravated by iNKT cell deficiency. TEM showed that the mitochondrial cristae of Sham mice were dense and clear, the mitochondria of WT-SP mice were swollen, the cristae were reduced or broken, the number of mitochondria was significantly increased, and the surface density and volume density of mitochondrial cristae were significantly decreased. Compared with WT-SP mice, the mitochondria of KO-SP mice were severely vacuolated, the cristae disappeared completely, and even the membrane ruptured. The number of mitochondria was further increased, and the surface density of mitochondrial cristae and mitochondrial volume density were further reduced (Fig.5E–G). Moreover, the mtProtein and mtDNA contents were markedly decreased in SP mice (Fig.5H–I), and the activities of ATP and mitochondrial complex enzyme I, II, and III were also significantly decreased (Fig.5J–M). The decreased level of KO-SP mice was the most obvious, and the above indexes decreased the least. In terms of oxidative stress indicators, the DHE fluorescence level and MDA content of WT-SP and KO-SP mice increased significantly, and the SOD content decreased significantly. The ROS and MDA levels of KO-SP mice were the highest, and the SOD activity was the lowest (Fig.5N–Q). In summary, iNKT cell defects trigger ROS generation by disrupting mitochondrial calcium homeostasis, leading to severe oxidative stress and energy metabolism disorders, and aggravating atrial mitochondrial damage in the SP model.

### iNKT deficiency further up-regulated IL-6/STAT3 signaling protein on SP mice

**Fig. 6:**
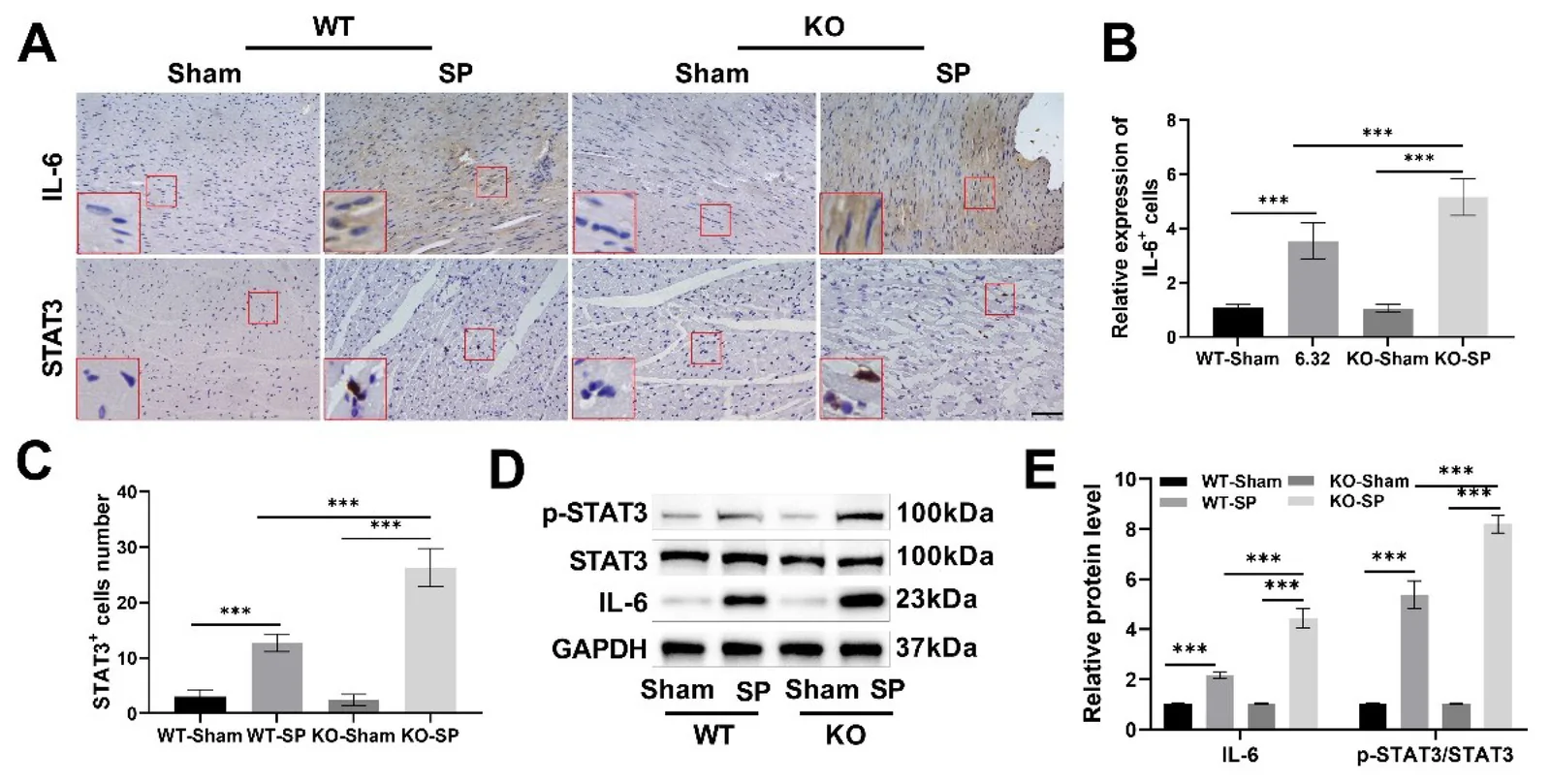
iNKT deficiency further up-regulated IL-6/STAT3 signaling protein in SP mice. **A–C**) IL-6 and STAT3 expressions on atrial tissue were assayed by immunohistochemistry, which was significantly increased in SP mice (×40, 50 μm). **D–E**) Western blot detected IL-6/STAT3 signaling protein. IL-6 and p-STAT3 protein expressions were markedly enhanced in SP mice. n=7, **P < 0.01, ***P < 0.001.

This study explored the potential signaling pathways of iNKT cell defects aggravating SP. Compared with Sham mice, IL-6 and STAT3 positive cell numbers in SP atrial tissues was significantly increased, and the number was the highest in KO-SP mice (Fig. 6A–C). IL-6 and p-STAT3 protein expressions in KO-SP mice was the highest among all mice (Fig.6D–E). In conclusion, SP activated IL-6/STAT3 axis within atrial tissue, and iNKT cell defect resulted in further activation of this pathway.

### Anti-IL-6 neutralizing antibody improved AF susceptibility in iNKT-deficient SP mice via inhibiting IL-6/STAT3 axis

**Fig. 7:**
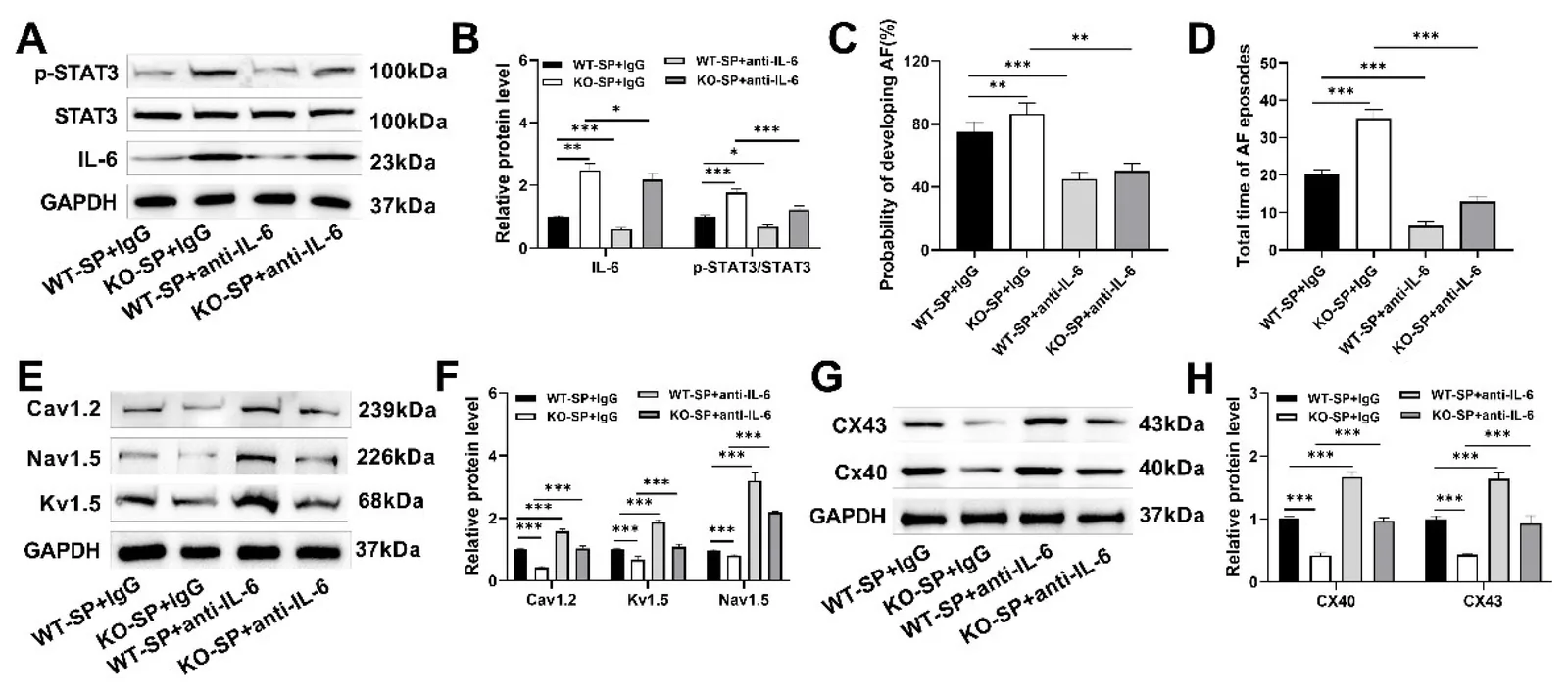
Anti-IL-6 neutralizing antibody improved AF susceptibility in iNKT-deficient SP mice *via* suppressing IL-6/STAT3 axis. **A–B**) Western blot detected IL-6/STAT3 signaling protein. Anti-IL-6 significantly reduced IL-6 and p-STAT3 protein levels. **C–D**) The statistical data of the probabilities and durations of AF induced 2 days after surgery were significantly reduced after anti-IL-6 intervention. **E–H**) AF-related proteins in atrial tissue were detected using western blot. Anti-IL-6 significantly increased the levels of Cav1.2, Kv1.5, Nav1.5, CX40, and CX43 proteins. n=7, *P < 0.05, **P < 0.01, ***P < 0.001.

Verifying the central roles of IL-6/STAT3 signaling in iNKT cell defects aggravating AF susceptibility, we used anti-IL-6 neutralizing antibody (anti-IL-6) for intervention at 36 h after SP induction and compared it with isotype control antibody (IgG). The data showed that in SP mice, anti-IL-6 significantly reduced IL-6 and p-STAT3 protein compared with IgG (Fig.7A–B), demonstrating that anti-IL-6 successfully blocked the excessive activation of IL-6/STAT3 axis. Moreover, anti-IL-6 also significantly reduced AF induction rate and AF duration (Fig. 7C–D), and increased Cav1.2, Kv1.5, Nav1.5, CX40, and CX43 protein levels (Fig.7E–H). The above results indicated that iNKT cell defects aggravated AF susceptibility in SP mice through hyperactivation of IL-6/STAT3 axis, and anti-IL-6 targeted inhibition of IL-6/STAT3 pathway could effectively reverse harmful electrophysiological remodeling, thereby significantly reducing AF susceptibility.

### Anti-IL-6 neutralizing antibody improved atrial tissue mitochondrial dysfunction, inflammation and fibrosis in iNKT-deficient SP mice by inhibiting IL-6/STAT3 pathway

**Fig. 8:**
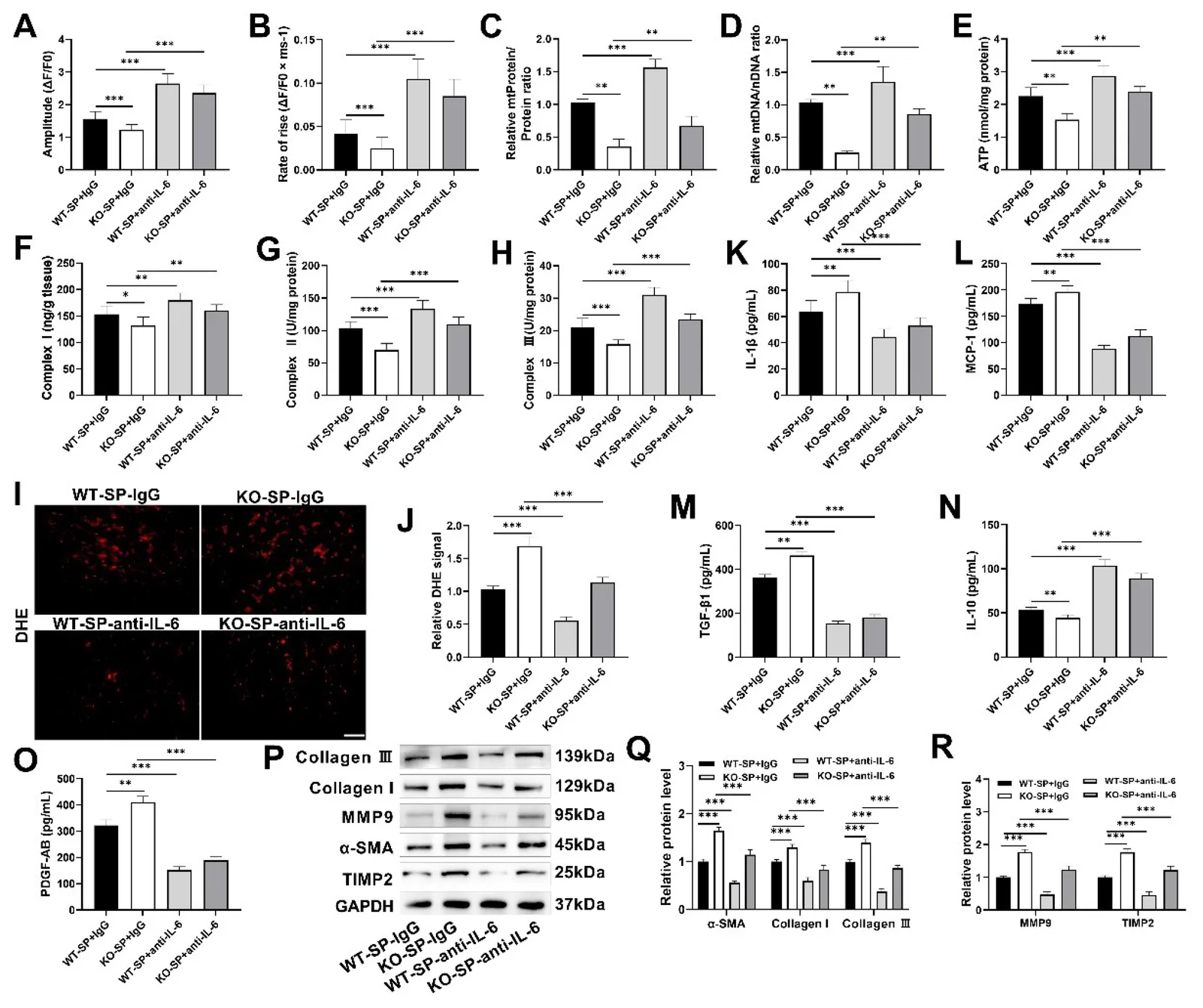
Anti-IL-6 neutralizing antibody improved atrial tissue mitochondrial dysfunction, inflammation and fibrosis in iNKT-deficient SP mice by inhibiting IL-6/STAT3 axis. **A–B**) Transient parameters of Ca^2+^ in atrial tissue were calculated, and anti-IL-6 significantly increased the amplitude and rate of Ca^2+^ increase. **C–D**) The content of mtProtein and mtDNA in atrial tissue was detected, which was significantly increased after anti-IL-6 intervention. **E–H**) ATP content and mitochondrial complex enzyme I, II, III activity in atrial tissue were measured, which were significantly increased after anti-IL-6 intervention. **I–J**) DHE staining assessed the level of ROS in atrial tissue, which was significantly reduced after anti-IL-6 intervention (×40, 50 μm). **K–O**) ELISA detected inflammatory factors. Anti-IL-6 significantly reduced the contents of IL-1β, IL-6, MCP-1, TGF-β1, and PDGF-AB, and increased IL-10 level. **P–R**) The expression of fibrosis protein in atrial tissue was assessed using western blot. α-SMA, Collagen I, Collagen III, MMP9, and TIMP2 expressions were significantly decreased after anti-IL-6 intervention. n=7, *P < 0.05, **P < 0.01, ***P < 0.001.

This study clarified anti-IL-6 protective effect on atrial tissue in SP mice, which was evaluated from three dimensions: mitochondrial function, inflammation level and fibrosis degree. In SP mice, anti-IL-6 markedly enhanced the transient amplitude and rising rate of mitochondrial Ca^2+^ (Fig. 8A–B), increased the content of mtProtein, mtDNA, ATP, and the activity of mitochondrial complex enzyme I, II, and III (Fig.8C–H), and decreased the level of ROS (Fig.8I–J). This indicated that inhibiting IL-6/STAT3 axis effectively improved mitochondrial calcium homeostasis and dysfunction in SP mice and reduced oxidative stress. ELISA showed that anti-IL-6 significantly reduced the content of IL-1β, IL-6, MCP-1, TGF-β1, and PDGF-AB, and elevated IL-10 content (Fig.8K–O). Additionally, anti-IL-6 significantly reduced α-SMA, Collagen I, Collagen III, MMP9, and TIMP2 protein levels (Fig.8P–R). This indicated that anti-IL-6 effectively inhibited atrial inflammation and fibrosis by inhibiting IL-6/STAT3 pathway. In summary, anti-IL-6 significantly improved atrial homeostasis in iNKT cell-deficient SP mice by inhibiting IL-6/STAT3 axis. Therefore, one of atrial homeostasis mechanisms in iNKT cell-deficient SP mice was mediated by IL-6 and downstream STAT3.

## Discussion

Despite the continuous progress and improvement of cardiac surgery technology and perioperative management, the incidence of POAF in cardiac surgery is still high. Due to the difficulty in obtaining atrial specimens from POAF patients, the mechanism of POAF is often studied by constructing SP models. iNKT cells identify glycolipid antigens in a CD1d-dependent manner and have a major role in immune regulation [23]. The critical role and molecular mechanism of iNKT cell defects in the occurrence of POAF were revealed for the first time through POAF mouse model. Defects in iNKT cells can induce mitochondrial calcium homeostasis imbalance and dysfunction via activating IL-6/STAT3 axis, exacerbating local inflammation and fibrosis in atrial tissue, causing a marked increase in AF susceptibility. This finding offers a new perspective for understanding POAF immune metabolism mechanism.

Although previous studies have confirmed that immune cells such as T lymphocytes and macrophages infiltrate and are vital for AF [24, 25], the specific role of iNKT cells is still unclear. NKT cells are a unique subset of T lymphocytes and classical NKT cells. They express not only the surface markers of T cells, but also the surface markers of NK cells CD161 (NK1.1) and NK cell receptor P1C. iNKT cells usually recognize lipid antigens presented by CD1d molecules on the surface of antigen-presenting cells, have phagocytic cytotoxicity and secretion, and are vital for rapid antigen recognition, initiation of immune response and regulation of other immune cell activity [26]. The stimulation of T lymphocytes leads to the increased expression of various surface antigens, which correspond to distinct phases of activation. CD69 serves as the most immediate indicator of T cell activation and functions as a costimulatory signal that enhances cytotoxic activity [27]. In this study, by using wild-type mice and SP model, the numbers of CD1d positive leukocytes, iNKT cells, and CD69^+^ cells within atrial tissue of SP mice was significantly increased, indicating that iNKT cells were up-regulated in POAF mice. These experiments showed that SP could significantly change the numbers of iNKT cells in atrial tissues, so iNKT cells might be involved in POAF. This suggests that iNKT cells may be the key guardian to maintain cardiac immune homeostasis.

In this study, Jα18^−/−^ mice (iNKT cell defect model) were used to establish SP model, and AF was induced by S1S2 stimulation on the second day after operation. Data showed that iNKT cell defects significantly increased the induction rate and duration of AF, showing more frequent atrial ectopic or fibrillation. From the perspective of electrophysiology, AF is caused by the abnormal expression or function of sodium, potassium, calcium, and other ion channels, which causes the rise of atrial asynchronous depolarization frequencies, the loss of atrial effective contraction and the irregularity of ventricular rate. Conduction block and reentrant are vital for the change of cardiac activity in AF, which is closely related to the gap junction protein between atrial myocytes. Connexins form gap junctions between adjacent cardiomyocytes, which provide electrical coupling and facilitate the propagation of action potentials. Among them, CX40 and CX43 are most closely related to AF [28–30]. The expression levels of ion channels (Cav1.2, Kv1.5, and Nav1.5) and gap junction proteins (CX40 and CX43) were significantly decreased in SP mice with iNKT cell defects, suggesting impaired electrical stability of the atrial tissue, including altered action potential duration and slowed conduction velocity, which predisposes the heart to reentrant excitation and fibrillation. This study speculates that iNKT cell defects will change ion channels, affect the localization and expression of connexins, and mediate AF maintenance. Therefore, iNKT cells play a key protective role in maintaining atrial electrical homeostasis.

Recent studies have found that cardiac inflammatory cells can cause arrhythmias by changing tissue composition or interacting with cardiomyocytes [31]. Inflammation is an important biological process in mammals to maintain the balance of the body. It is the physiological response of the body to its own infection and injury. However, under pathological conditions, excessive inflammatory factors will change the permeability of blood vessels, so that inflammatory cells and inflammatory factors migrate to target organs such as the heart, leading to atrial electrical remodeling and structural remodeling, and promoting AF [31–33]. Inflammation can lead to myocardial cell necrosis, recruitment of inflammatory cytokines and activation of cardiac fibroblasts, leading to myocardial structural remodeling and fibrosis [34]. During fibrosis, due to excessive activation of myocardial fibroblasts and increased extracellular matrix, local conduction block or reentry is caused, resulting in uneven electrical conduction of myocardial tissue and providing matrix conditions for the occurrence of arrhythmia [35]. In conclusion, atrial inflammation and fibrosis are the core factors for the occurrence and persistence of AF [4]. Du et al. showed that circNAB1 reduces AF by reducing inflammation and fibrosis [36]. Parent et al. found that AF can be prevented by reducing pro-inflammatory factors in atrial tissue and reducing atrial fibrosis [1]. In this study, a large number of inflammatory cell infiltration, cardiomyocyte cytolysis and necrosis, and blue collagen deposition were observed in the atrial tissue of iNKT cell-deficient SP mice. Inflammatory factors (IL-1β, IL-6, MCP-1, TGF-β1, and PDGF-AB) and fibrotic proteins (α-SMA, Collagen, MMP9, and TIMP2) were markedly enhanced, indicating that iNKT cell deficiency aggravated atrial inflammation and fibrosis in POAF. It should be noted that in the present study, the observed ‘fibrosis’ at the 48-hour time point primarily manifested as inflammatory fibrous tissue proliferation on the epicardium/pericardium and early fibroblast activation, characterized by increased α-SMA and Collagen synthesis, rather than established myocardial interstitial fibrosis.

Calcium ions serve as key regulators in mitochondrial metabolism. A previous study has found that the imbalance of Ca^2+^ homeostasis is associated with AF. Mitochondrial energy metabolism disorder is involved in the occurrence of AF through the Ca^2+^ signal-dependent pathway in atrial myocytes [37]. It has been reported that mitochondrial energy metabolism disorders can lead to insufficient ATP production and excessive ROS, thereby disrupting the homeostasis of Ca^2+^ in cardiomyocytes and membrane excitability leading to calcium overload and further damage, leading to the occurrence of AF [38]. An excess of Ca^2+^ promotes the generation of ROS by stimulating electron donation to the electron transport chain while simultaneously impairing the function of complexes I and III [39]. This rise in ROS levels then leads to the oxidation of Ca^2+^-sensitive proteins, including RyR2, resulting in their hyperactivation and the consequent dysregulation of cellular calcium balance [40]. In addition, in Ca^2+^-overloaded mitochondria, electron transport efficiency is reduced, resulting in increased superoxide production and decreased ATP synthesis [41]. Studies have shown that mtDNA is involved in the pathogenesis of AF, mainly including structural remodeling, electrical remodeling, and ion channel abnormalities. The above studies have shown that patients with AF are accompanied by mitochondrial dysfunction, imbalance of Ca^2+^ homeostasis and electrical remodeling. Hamilton et al. proved that the release of endoplasmic reticulum Ca^2+^ induced by mitochondrial Ca^2+^ concentration was achieved by mitochondrial ROS modifying RyR2, thereby aggravating arrhythmia [42]. Zhang *et al*. found that succinate damaged the structure and function of atrial mitochondria, leading to increased oxidative stress, increased AF susceptibility and adverse ventricular remodeling, which was characterized by left atrial dilatation, connexin lateralization, ion channel disorder and fibrosis [43]. Similar to previous research findings, the transient amplitude and rising rate of mitochondrial Ca^2+^ were significantly reduced in the atrial tissue of SP mice with iNKT cell deficiency. Severe vacuolization of mitochondria, complete disappearance of cristae, even membrane rupture, increased number of mitochondria; the contents of mtProtein, mtDNA, and ATP, and the activity of mitochondrial complex enzyme were significantly decreased, while the ROS content and p-RyR2 expression level were significantly increased. This study speculated that under SP stress, mitochondrial calcium overload in atrial tissue, which in turn triggered ROS outbreak, causing severe oxidative stress, which further damaged the functions of mitochondrial electron transport chain, resulting in energy synthesis disorder (ATP deficiency), and ultimately manifested as the destruction of mitochondrial structure and quantity. The defect of iNKT cells aggravates the above links, indicating that iNKT cells may be crucial for cardiac protection by stabilizing mitochondrial calcium homeostasis and reducing oxidative stress.

IL-6 is a multi-effect cytokine involved in immune response, inflammation and other processes [44]. STAT3 is vital for pathological processes such as inflammation, cardiovascular disease, and tumor progression [45]. STAT3 is the downstream target of IL-6. IL-6 activates IL-6/STAT3 axis by phosphorylating STAT3, then participates in inflammatory response process. This pathway is activated and aggravates the process of cardiac injury induced by angiotensin II [46]. The increased expression of IL-6 in myocardium was linked to doxorubicin (DOX)-induced cardiac injury [47]. Bone morphogenetic protein 10 attenuated cardiac dysfunction and cardiac injury by regulating STAT3 signaling pathway [48]. Studies have suggested that IL-6 is strongly associated with POAF occurrence. IL-6 also has a significant effect on the imbalance of atrial calcium homeostasis, and the treatment of IL-6 antibody significantly alleviates the imbalance of calcium homeostasis in SP rats and reduces the susceptibility of AF [49]. This study also found that SP activated IL-6/STAT3 axis, and iNKT cell defect would lead to further activation of this pathway. Intervention with IL-6 neutralizing antibody can effectively reverse AF susceptibility, atrial remodeling, mitochondrial dysfunction, inflammation and fibrosis caused by iNKT cell defects.

## Conclusion

In summary, iNKT cell deficiency can significantly aggravate susceptibility to AF, inflammation, fibrosis, and mitochondrial dysfunction in POAF, and its mechanism is achieved via IL-6/STAT3 axis. This finding not only reveals the regulatory mechanism of iNKT cells in POAF immunity, but also provides new ideas and experimental basis for precise immunotherapy strategies based on iNKT cells. Unfortunately, this study only conducted a preliminary exploration at the animal level, and the next step will be to demonstrate the results from clinical samples. And this study relies on a mouse model that may not fully simulate the pathological conditions of human POAF.

## Supplementary Information



## Figures and Tables

**Table 1:** Primary antibodies

Protein name	Dilution ratio	Catalog number	Producer
*cluster of differentiation 1d (CD1d)*	1 : 1 000	ab215445	Abcam
*Cav1.2*	1 : 1 000	ab270987	Abcam
*Kv1.5*	1 : 1 000	DF4313	Affinity
*Nav1.5*	1 : 1 000	ab300048	Abcam
*connexin 40 (CX40)*	1 : 1 000	ab313644	Abcam
*connexin 43 (CX43)*	1 : 1 000	ab314908	Abcam
*α-smooth muscle actin (α-SMA)*	1 : 5 000	BF9212	Affinity
*Collagen I*	1 : 5 000	ab316222	Abcam
*Collagen III*	1 : 1 000	ab184993	Abcam
*matrix metalloproteinase 9 (MMP9)*	1 : 1 000	ab283575	Abcam
*tissue inhibitor of metalloproteinases-2 (TIMP2)*	1 : 2 000	ab180630	Abcam
*ryanodine receptor 2 (RyR2)*	1 : 1 000	ab219798	Abcam
*p-RyR2*	1 : 2 000	AF7454	Affinity
*inositol 1,4,5-trisphosphate receptor type 1 (IP3R1)*	1 : 10 000	ab264281	Abcam
*voltage-dependent anion channel 1 (VDAC1)*	1 : 1 000	ab306581	Abcam
*glucose-regulated protein 75 (GRP75)*	1 : 3 000	ab227215	Abcam
*IL-6*	1 : 1 000	ab290735	Abcam
*signal transducer and activator of transcription 3 (STAT3)*	1 : 10 000	ab109085	Abcam
*p-STAT3*	1 : 1 000	ab219593	Abcam
*GAPDH*	1 : 50 000	AF0911	Affinity

## Abbreviations

α-SMA: α-smooth muscle actin
AF: atrial fibrillation
ATP: adenosine triphosphate
CD1d: cluster of differentiation 1d
CX40: Connexin 40
CX43: Connexin 43
GRP75: glucose-regulated protein 75
IFN-γ: interferon-γ
IL: interleukin
iNKT: invariant natural killer T
IP3R1: inositol 1,4,5-trisphosphate receptor type 1
MCP-1: monocyte chemoattractant protein-1
MDA: malondialdehyde
MMP9: matrix metalloproteinase 9
MPO: myeloperoxidase
mtDNA: mitochondrial DNA
mtProtein: mitochondrial protein
NK: natural killer
NKT: natural killer T cells
PDGF-AB: platelet-derived growth factor AB
POAF: postoperative atrial fibrillation
ROS: reactive oxygen species
RyR2: ryanodine receptor 2
SOD: superoxide dismutase
SP: sterile pericarditis
STAT3: signal transducer and activator of transcription 3
TAC: tricarboxylic acid cycle
TCR: T cell receptor
TGF-β1: transforming growth factor β1
TIMP2: tissue inhibitor of metalloproteinases-2
VDAC1: voltage-dependent anion channel 1
