# Supplementary material for: The Defect of iNKT Cells Mediates Mitochondrial Dysfunction Through IL-6/STAT3 Signaling to Induce Atrial Inflammation and Fibrosis to Aggravate Postoperative Atrial Fibrillation
Source: Physiol Res. 2026 Aug 1;75(4):679–97. doi: 10.33549/physiolres.935803 (PMC13580675; doi:10.33549/physiolres.935803)
Supplement: Supplementary file 1 [file PR75_679_TableS1.pdf]

Table S1 ECG parameters (n = 7)

	WT-Sham	WT-SP	KO-Sham	KO-SP
RR (ms)	123.65±4.45	126.49±2.87	124.44±4.67	127.73±4.02
P wave duration (ms)	12.03±0.02	12.00±0.03	12.10±0.02	12.04±0.02
PR (ms)	43.9±0.61	44.15±1.01	43.04±0.85	45.1±0.69
QRS (ms)	15.7±0.33	14.5±0.2	16.3±0.6	14.9±0.4
SNRT100 (ms)	142±8.28	144±9.45	143±8.04	147±6.12
AERP100 (ms)	48.6±1.25	49.8±1.37	50.3±1.62	48.9±1.30
